# Inhibition of growth rate and cylindrospermopsin synthesis by *Raphidiopsis raciborskii* upon exposure to macrophyte *Lemna trisulca* (L)

**DOI:** 10.1007/s10646-021-02377-7

**Published:** 2021-03-12

**Authors:** Kornelia Duchnik, Jan Bialczyk, Ewelina Chrapusta-Srebrny, Beata Bober

**Affiliations:** Department of Plant Physiology and Development, Jagiellonian University, Gronostajowa 7, 30-387 Kraków, Poland

**Keywords:** Allelopathy, Biomass accumulation, Cylindrospermopsin, *Lemna trisulca*, *Raphidiopsis raciborskii*

## Abstract

Impact of macrophyte *Lemna trisulca* on the growth rate and synthesis of cylindrospermopsin (CYN) by cyanobacterium *Raphidiopsis raciborskii* was determined. The presence of *L. trisulca* inhibited the biomass accumulation of the cyanobacterium by 25% compared to the control during co-cultivation. The simultaneous cultivation of these organisms slightly affected the inhibition of macrophyte growth rate by 5.5% compared to the control. However, no morphological changes of *L. trisulca* after incubation with cyanobacteria were observed. It was also shown that the long-term (35 days) co-cultivation of *R. raciborskii* and *L. trisulca* led to a decrease in CYN concentration in media and cyanobacterial cells by 32 and 38%, respectively, compared to the values obtained for independent cultivation of cyanobacterium. Excessive absorption of phosphate ions by *L. trisulca* from the medium compared to nitrate ions led to a significant increase in the nitrate:phosphate ratio in the media, which inhibits the development of cyanobacterium. The obtained results indicate that *L. trisulca* in the natural environment may affect the physiology of cyanobacteria. The presented study is the first assessment of the allelopathic interaction of macrophyte and *R. raciborskii*.

## Introduction

In aquatic ecosystems, macrophytes and phytoplankton represent a group of primary producers. Macrophytes play an essential role in the nutrient cycling in the environment through their significant accumulation in submerged roots or leaves, thus limiting the availability of necessary elements for the development of phytoplankton (Pott and Pott 2003). They also provide shelter for invertebrates, macrophages, zooplankton and young fish (Mulderij et al., 2007). The metabolism of macrophytes affects the physicochemical properties of water, including changes in concentration of oxygen, inorganic carbon or pH (Caraco and Cole, 2002). Chemical interactions between aquatic organisms attract the attention of researchers from around the world (Pflugmacher, 2002). It is believed that using macrophytes might be an effective, safe and ecofriendly method for controlling and removing toxic cyanobacterial blooms from the water.

So far, numerous studies have been carried out on the effects of allelopathic compounds released by macrophytes into the water environment on cyanobacteria (Gross, 1999; Hong et al., 2011; Nakai et al., 2012, Mohamed 2017). Some of them, such as tellimagrandin II, pyrogallic or gallium acids synthesized by *Myriophyllum spicatum* significantly impair the photosynthetic activity of *Anabaena* sp. and *Microcystis aeruginosa* (Leu et al., 2002; Zhu et al., 2010). Oxidative damage, including loss of membrane integrity and inhibition of protein synthesis, is also an important mechanism of toxicity of plant allelochemicals against *M. aeruginosa* (Lu et al., 2016; Tazart et al., 2020). However, allelopathic products can simultaneously stimulate a significant increase in the activity of antioxidant enzymes, e.g., superoxide dismutase and catalase, suggesting their involvement in the elimination of oxidative stress in cyanobacteria (Hong et al., 2008; Lu et al., 2016; Zhang 2015; Hua et al., 2018; Kang et al., 2020; Tazart et al., 2020). Additionally, various phenolic compounds, fatty acids, terpenoids and asarone synthesized by aquatic plants reduce the biomass of cyanobacteria and induce changes in their cell and colonial morphology (Nakai et al., 2000; Rojo et al., 2013; Hang, 2017; Mowe et al., 2019; Tazart et al., 2019).

Although *Raphidiopsis raciborskii* is the main producer of the cytotoxic cylindrospermopsin (CYN) documented in water bodies all over the world (Rzymski and Poniedziałek 2014; Kleinteich et al., 2014), there is no information in the literature on the allelopathic effect of macrophytes occupying the same ecological niche on its physiology and biochemistry. CYN, in animal organisms, inhibits protein synthesis, causes damage to cell nuclei, increases the concentrations of reactive oxygen species, induces DNA fragmentation (Lankoff et al., 2007) as well as affects organs such as liver (Terao et al., 1994) and kidneys (Norris et al., 2001). An additional risk is the accumulation of CYN in the food chain that can pose a great threat to all organisms, including humans. This explains the growing interest in recent years in searching for methods to reduce *R. raciborskii* blooms and thus eliminate CYN from the environment.

Therefore, *Lemna trisulca*, which occurs in the natural environment in direct contact with cyanobacterium and cyanotoxin was selected for the study. This macrophyte effectively reduces the development of other cyanobacterium, *Anabaena flos-aquae*, and the concentration of synthetized anatoxin-a (Kaminski et al., 2015), what additionally confirms this choice. In order to assess the influence of *L. trisulca* on limiting the development of *R. raciborskii* and the level of CYN, we examined changes in biomass accumulation, concentrations of nitrogen and phosphorus in media and synthesis of cylindrospermopsin by cyanobacterium during long-term cultivation of these two organisms. It is assumed that the results can be used in the future to reduce the formation of cyanobacterium blooms in water bodies and to bioremediate the produced toxin.

## Materials and methods

### Growth conditions of the test organisms

To the experiments we used the cultures of *Raphidiopsis raciborskii* (Wołoszyńska) Aguilera, Berrendero Gómez, Kaštovský, Echenique & Salerno *comb. nov*. strain CS-505/7 obtained from the Australian Algae Culture Collection (Australia) and *Lemna trisulca* strain LT-FR 1 collected from the natural environment and brought into pure, sterile culture in our laboratory. Both organisms were cultivated in separate Erlenmeyer flasks containing BG-11 medium (Rippka et al. 1979) in a phytotron (Bolarus S-711S/P, Poland) at 20 ± 1 °C, with 80% humidity under 50 μmol m^−2^ s^−1^ photosynthetically active radiation (PAR) with a light/dark period of 12/12 h. Each flask was shaken regularly every day.

### The experimental procedure

The biweekly *R. raciborskii* cultures were transferred to 50 mL of falcons and centrifuged (10,000 *g* × 5 min). Cyanobacterial cells were then suspended in a small volume of fresh medium and shaken on SK-0330-PRO shaker (Chemland, Poland). The concentrated cell suspension of 3 mL was added to the 100 mL sterile Erlenmeyer flasks filled with 40 mL of aerated BG-11 medium for cultivation of (a) cyanobacterium, and (b) cyanobacterium with macrophyte. To the latter ones as well as to the flasks planned for the cultivation of the macrophyte itself, 1 g fresh weight (fw) of *L. trisulca* was added. Each experimental series was prepared in five independent replicates. The initial ratio of the dry weight of macrophyte and cyanobacterium was 26: 1 (w/w). The experiment was conducted under the physical conditions described above, and the samples were taken in 5, 10, 15, 20 and 35 day of cultivation. A specially prepared set was used to separate the medium, cyanobacterium and macrophyte after cultivation. It was consisted of: a vacuum flask connected to a vacuum pump, a Büchner funnel with Whatman’s previously weighed filter and a 2 mm metal sieve. During the separation of the two-species culture, first *L. trisulca* was stopped on the strainer, then *R. raciborskii* cells were accumulated on Whatman’s filter, and finally, the medium was collected in the flask. After separation of all components, the set was rinsed with 10 mL of Milli Q water for removal of unbound CYN from the surface of the plant and cyanobacterial cells.

### Measurements

#### Biomass accumulation

*L. trisulca* and *R. raciborskii* were subjected to a freeze-drying process to obtain a dry weight. The dry weight of cyanobacterium was determined using the weight of empty Whatman’s filter and respective that with embedded cyanobacteria cells.

#### Concentration of CYN

CYN was extracted and purified from the cyanobacteria cells, media and plant tissues as described by Meriluoto and Codd (2005) with some modifications. The toxin was quantified using a High-Performance Liquid Chromatography (HPLC) Waters system consisting of a 600E gradient pump, a 717 plus autosampler, a 996 photodiode array (PDA) detector, a Millenium^32^ SS Software with PDA option, a Jetstream 2 plus column thermostat and a reverse phase column Phenomenex Gemini^®^ (250 × 4.6 mm; 5 µm) kept at 35 °C. The PDA range was 210–400 nm with a fixed wavelength of 262 nm. The gradient mobile phase consisted of water/acetonitrile (both with 0.05% trifluoroacetic acid) changed from 100 to 70% water in 20 min at a flow rate of 1.0 mL min^−1^. CYN was identified and quantified by comparing of the UV-spectra determined for the standard.

#### pH determination

Values of media pH were determined using a glass pH microelectrode (InLab 423, Mettler Toledo, Switzerland).

#### Concentration of nitrate and phosphate ions

The concentration of nitrate and phosphate ions in media were carried out with using the DX600A ion chromatograph (Dionex, USA) containing AS40 autosampler and ED50 detector, the AS9-HC column (4 × 250 mm) and a liquid phase consisting of a 9 mM solution of Na_2_CO_3_. The PeakNet program was used for analyzing results.

#### Chemicals

All reagents used for the experiment were purchased from Sigma-Aldrich (St. Louis, MO, USA). The commercial standard of CYN (purity > 95%) was supplied from Sigma-Aldrich (St. Louis, MO, USA). Ultrapure grade water (Milli-Q water) was obtained from Millipore (Bradford, MA, USA).

### Statistical analysis

The reported data were expressed as the mean ± SD of five replicates. All the obtained results were subjected to one-way or two-way ANOVA, and all the means were tested for significant differences (*p* < 0.05) with Tukey’s test. Statistical analyses were made using STATISTICA 13 software.

## Results

### Biomass accumulation

**Fig. 1:**
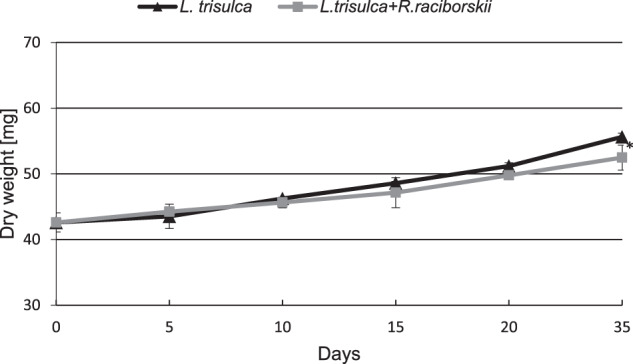
Changes of the dry weight of *L. trisulca* cultivated individual or with *R. raciborskii*. Data are expressed as mean ± SD (*n* = 5), *significant difference from control at *p* < 0.05

**Fig. 2:**
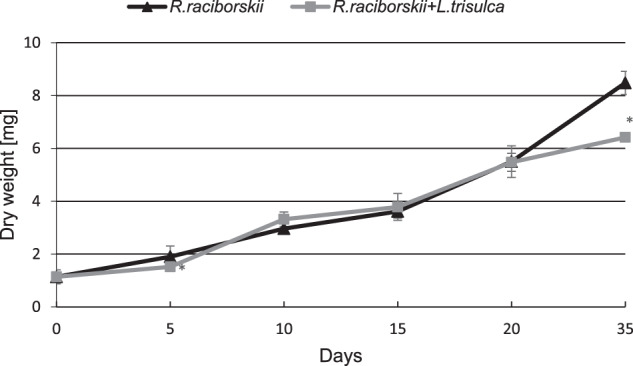
Changes of the dry weight of *R. raciborskii* cultivated individual or with *L. trisulca*. Data are expressed as mean ± SD (*n* = 5), *significant difference from control at *p* < 0.05

Short-term (up to 10 days) simultaneous cultivation of cyanobacterium and macrophyte did not affect the plant’s biomass accumulation compared to the control (*F* = 0.35, *p* = 0.56). After 20 and 35 days of co-cultivation, a decrease in *L. trisulca* biomass accumulation by 3% (*F* = 1.95, *p* = 0.18) and 5.5% were shown (*F* = 6.79, *p* = 0.02), respectively (Fig. 1). During the 20 days of the experiment, no statistically significant changes in the growth of the cyanobacterial biomass were observed (*F* = 0.06, *p* = 0.81). However, on day 35 of cultivation the presence of *L. trisulca* inhibited the development of *R. raciborskii*. The final dry weight of cyanobacterial cells represented 75% of the value determined for independent cultivation (*F* = 123.72, *p* < 0.05) (Fig. 2).

### CYN concentration in the medium, plant tissues and cyanobacterial cells

**Fig. 3:**
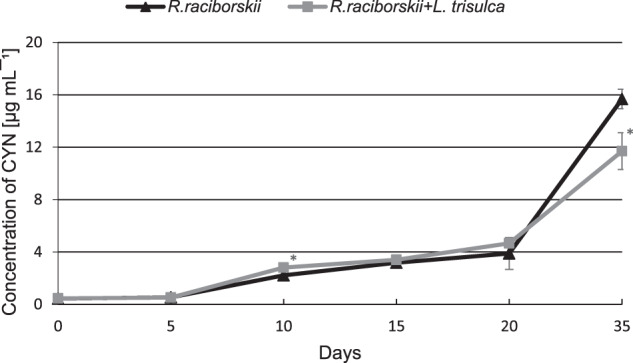
The concentration of CYN in the medium during 35 days of independent cultivation of *R. raciborskii* or simultaneous with *L. trisulca*. Data are expressed as mean ± SD (*n* = 5), *significant difference from control at *p* < 0.05

**Fig. 4:**
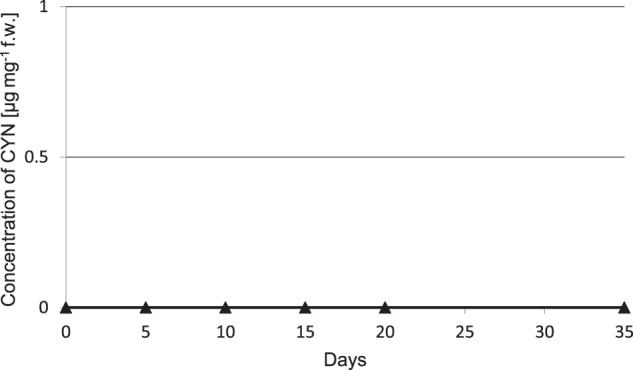
The concentration of CYN in *L. trisulca* tissues during 35 days cultivation with *R. raciborskii*. Data are expressed as mean ± SD (*n* = 5), *significant difference from control at *p* < 0.05, *fw* fresh weight

**Fig. 5:**
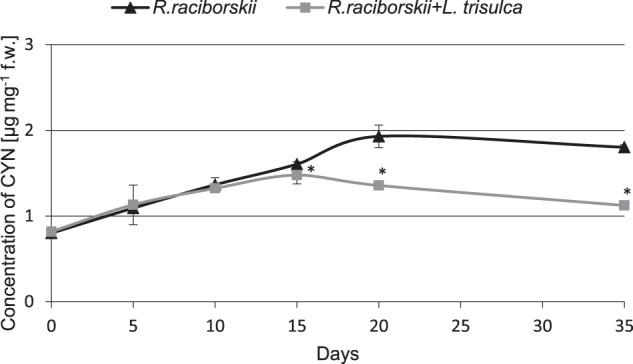
The concentration of CYN in cyanobacterial cells during 35 days of independent cultivation of *R. raciborskii* or simultaneous with *L. trisulca*. Data are expressed as mean ± SD (*n* = 5), *significant difference from control at *p* < *0.05, fw* fresh weight

The initial CYN concentration in the medium was 0.4 µg mL^−1^. After 35 days of the experiments, toxin concentration in the medium of simultaneous cultivation reached only 68% of the value detected in the medium where cyanobacteria grew separately (*F* = 45.73, *p* < 0.05) (Fig. 3). Interestingly, no CYN was detected in *L. trisulca* tissues (Fig. 4). The initial concentration of CYN in *R. raciborskii* cells was 0.8 µg mg^−1^ fw and it increased in time. During the first 10 days, the changes in concentration level did not differ significantly between separate cultures of cyanobacterium and cyanobacterium with macrophyte (*F* = 2.73, *p* = 0.12) (Fig. 5). Then the CYN intracellular content in co-cultivating cyanobacterium decreased and on the 35th day was lower by 38% compared to separate cultivation of cyanobacterium (*F* = 924.53, *p* < 0.05).

### pH value in the medium

**Fig. 6:**
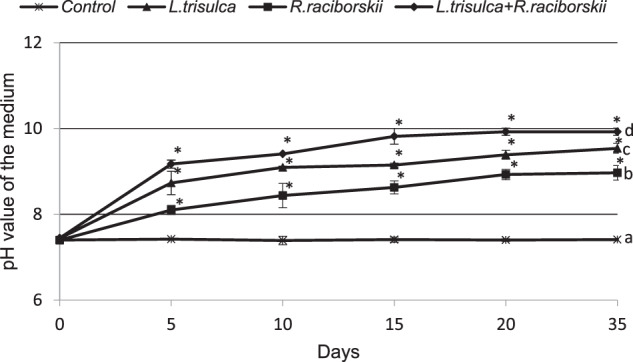
Changes of pH value of the medium over 35 days of cultivation of the control (without any organisms) (**a**), *R. raciborskii* (**b**), *L. trisulca* (**c**) and *L. trisulca* with *R. raciborskii* (**d**). Data are expressed as mean ± SD (*n* = 5), *significant difference from control at *p* < *0.05, fw* fresh weight

The initial pH value of 7.5 in the control medium (without organisms) did not change significantly during the whole experiment. The cultivation of *L. trisulca*, *R. raciborskii* and both organisms simultaneously stimulated the increase of the pH of the media during the experiment. In the experimental series tested, the highest pH values were found on the last day of the incubation. After 35 days, the pH value of the medium where *R. raciborskii*, *L. trisulca* or both organisms were cultivated increased by 1.46 (*F* = 855.91, *p* < 0.05), 2.00 (*F* = 3078.48, *p* < 0.05) or 2.50 units (*F* = 7953.73, *p* < 0.05), respectively (Fig. 6).

### Concentration of nitrate and phosphate ions in the medium

**Fig. 7:**
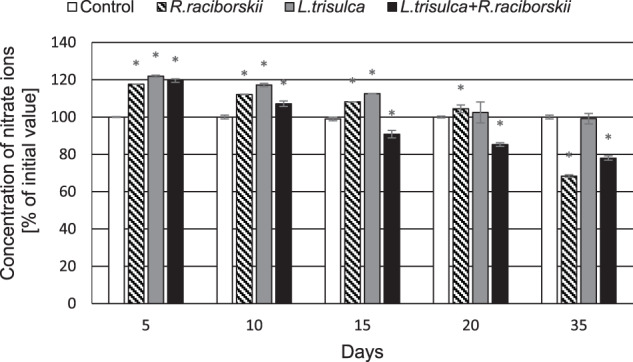
The concentrations of nitrate ions in the medium over 35 days of the experiment. Data are expressed as mean ± SD (*n* = 5), *significant difference from control at *p* < 0.05

**Fig. 8:**
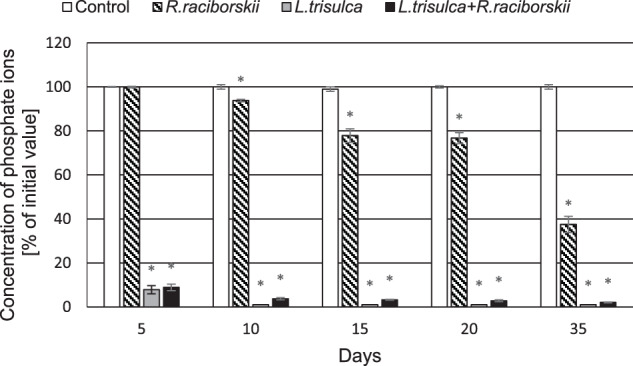
The concentrations of phosphate ions in the medium over 35 days of the experiment. Data are expressed as mean ± SD (*n* = 5), *significant difference from control at *p* < 0.05

Significant increase in the nitrate ions concentration by 20% compared to the initial value was demonstrated in medium of all experimental series after 5 days of cultivation (*F* = 1311.12, *p* < 0.05) (Fig. 7). On 35th day their concentration was determined at 100% (*F* = 0.19, *p* = 0.67), 68% (*F* = 6500.02, *p* < 0.05) and 78% (*F* = 2179.26, *p* < 0.05) of the initial value in the media after incubation of *L. trisulca, R. raciborskii* or *L. trisulca* with *R. raciborskii*, respectively. After just 5 days, the concentration of phosphate ions was reduced by 92% (*F* = 17224.53, *p* < 0.05) and 91% (*F* = 28783.57, *p* < 0.05) in media containing macrophyte or macrophyte with a cyanobacterium, respectively. In contrast, no significant change was detected in the medium containing cyanobacterium (*F* = 1.99, *p* = 0.20). Although this value decreased to 38% of the initial concentration on 35th day (*F* = 2320.24, *p* < 0.05), it was higher than determined for the other experimental series (Fig. 8).

## Discussion

### Biomass accumulation

The obtained results showed that the co-cultivation of *L. trisulca* with *R. raciborskii* resulted in a slight decrease in plant biomass accumulation compared to control samples (Fig. 1). Such insignificant changes could be associated with the depletion of minerals in the medium or the release of secondary metabolites synthesized by cyanobacteria into the external environment. Furthermore, the tested macrophyte did not show any visible morphological changes such as chlorosis or necrosis. The simultaneous 35 days cultivation of *L. trisulca* with *R. raciborskii* resulted in a 25% reduction of the growth rate of cyanobacterial biomass compared to the data obtained for independent cultivation of cyanobacterium (Fig. 2). The similar result was presented by Kaminski et al. (2015). Long-term (32 days) cultivation of *L. trisulca* with *Anabaena flos-aquae* caused a rapid decrease of the cyanobacterium growth rate and changes in the morphological structure of its cells. Jang et al. (2007) also demonstrated a significant reduction of *Microcystis aeruginosa* biomass during its cultivation with *L. japonica* compared to the values achieved for separate cyanobacterial culture. Moreover, Nakai et al. (1999) provided information on the inhibition of growth rate of several cyanobacterial species such as *M. aeruginosa*, *A. flos-aquae* and *Phormidium tenue* during simultaneous cultivation with macrophytes *Cobomba caroliniana* and *Myriophyllum spicatum*. The inhibition of cyanobacterial biomass accumulation may be a consequence of (a) an increase in pH value of the medium, (b) the appearance of a stress reaction caused by a deficiency of some ions, (c) an increase in the N:P ratio value, or (d) the synthesis of allelopathic compounds by macrophytes and their release into the medium. After long-term cultivation of *L. trisulca* with *R. raciborskii*, a very high level of CYN was determined in the media (11.7 µg mL^−1^) (Fig. 3), whereas the tested macrophyte did not show any visible morphological changes such as chlorosis or necrosis. This result suggests that during co-cultivation, macrophyte resistance to CYN produced by cyanobacterium is much higher than in conditions when a pure toxin is added into the medium (Flores-Rojas et al., 2015, Santos et al., 2015). The observed increase in plants resistance during simultaneous cultivation might be caused by (a) an additional production of other types of toxins by the tested cyanobacterial species that could exert an opposite effect to CYN (Holland and Kinnear, 2013), (b) the synthesis of various metabolites by cyanobacteria that regulate the macrophyte growth rate and eliminate the harmful effects of CYN, (c) the release of primary metabolites that the plant may have used, (d) the synthesis of plant secondary metabolites with a protective function, or (e) the potential effect of light shading by *L. trisulca* (Dziga et al., 2007; Hong et al., 2009). Kaminski et al. (2015) did not find significant differences in the macrophyte biomass accumulation during cultivation of *L. trisulca* with *A. flos-aquae* for 32 days and obtained similar results to ours. In contrast, opposite data were obtained by Jang et al. (2007), who after 12 days of *L. japonica* and *M. aeruginosa* co-cultivation demonstrated a 50% reduction in plant biomass compared to controls. The above results may suggest differences in the sensitivity of both *Lemna* species to the cyanobacterial toxins. Besides, the growth rate of *L. japonica* could be inhibited by microcystin (MC), a toxin produced by *M. aeruginosa* with a chemical structure and mechanism of action significantly different from CYN.

### Concentration of CYN in medium, plants tissues and cyanobacterial cells

It was shown that long-term (35 days) cultivation of *R. raciborskii* with *L. trisulca* led to a decrease in CYN concentration in the media and cyanobacterial cells compared to the values obtained for separate cultivation of cyanobacteria (Figs. 3 and 5). The reduction in toxin concentration in these media might be the result of an increase in the pH value to 10 (Fig. 6). This conclusion is supported by the documented decomposition of CYN under pH ≥10 (Chiswell et al. 1999; Adamski et al. 2016). Besides, in the media where the cyanobacterium was grown itself for a similar time, the pH increased only to 9. Inhibition of CYN synthesis by *R. raciborskii*, and thus the reduction of its amount in the cells after cultivation with *L. trisulca* might be associated with changes in the concentration of mineral components in the media due to the presence of macrophyte (Figs. 7 and 8). This process has been confirmed in a natural environment where the availability of minerals had a significant impact on the production of toxins by different types of cyanobacteria (Saker and Neilan 2001; Bar-Yosef et al., 2010). In contrast, Kearns and Hunter (2000) indicated that the presence of *Chlamydomonas reinhardtii* algae stimulated anatoxin-a (ANTX-a) synthesis by *A. flos-aquae* during their co-cultivation. Similar results were obtained by Jang et al. (2007), who demonstrated that 12 days cultivation of *L. japonica* with *M. aeruginosa* caused an increase in the intra- and extracellular concentration of MC. However, similarly to CYN, this toxin has not been identified in macrophyte tissues. Increased MC production likely exerted an allelopathic effect on the studied macrophyte by inhibiting its growth (Jang et al. 2007).

### Changes in the concentration of nitrate and phosphate ions

Excessive absorption of phosphate ions by *L. trisulca* (Fig. 8) compared to nitrate ions (Fig. 7) during cultivation with cyanobacterium led to a significant increase in the N:P ratio in the media. This was contributed to the effective inhibition of *R. raciborskii* growth rate after 35 days of the experiment. Furthermore, no changes in the N:P ratio were observed for independent cultivation of cyanobacteria. Similar results were shown by Kaminski et al. (2015). *L. trisulca* cultivated separately and together with *A. flos-aquae* caused a significant increase in the N:P ratio. However, in case of co-cultivation of *L. japonica* with *M. aeruginosa* such changes were not observed (Jang et al. 2007). Based on the above results, it can be assumed that *L. trisulca* in the natural environment affects the development and physiology of cyanobacteria.

## Conclusion

Cyanobacteria synthesizing toxic secondary metabolites in the aquatic environments constantly compete with other species of cyanobacteria, algae and macrophytes. The main role of cyanobacterial toxins in ecosystems is attributed to the function of the allelopathic interaction with other co-existing organisms (Holland and Kinnear, 2013). This is the first report presenting the results of interaction between *L. trisulca* and *R. raciborskii* during co-cultivation. Our results revealed a decrease in CYN production and growth rate inhibition of *R. raciborskii* upon long-term exposure to *L. trisulca*. It suggests that unknown compounds released by this macrophyte might perform the function as allelochemicals, which in the natural environment can effectively inhibit the development of toxic cyanobacterial blooms.

## Data Availability

All data generated during this study are included in the article.
